# Effectiveness of an Oral Health Education Program Using a Smart Toothbrush with Quantitative Light-Induced Fluorescence Technology in Children

**DOI:** 10.3390/children10030429

**Published:** 2023-02-22

**Authors:** Jihyun Lee, Taeyang Lee, Hoi-In Jung, Wonse Park, Je Seon Song

**Affiliations:** 1 Department of Pediatric Dentistry, College of Dentistry, Yonsei University, Seoul 03722, Republic of Korea; 2 Department of Preventive Dentistry & Public Oral Health, College of Dentistry, Yonsei University, Seoul 03722, Republic of Korea; 3 Innovation Research and Support Center for Dental Science, Yonsei University Dental Hospital, Seoul 03722, Republic of Korea; 4 Department of Advanced General Dentistry, College of Dentistry, Yonsei University, Seoul 03722, Republic of Korea; 5 Oral Science Research Center, College of Dentistry, Yonsei University, Seoul 03722, Republic of Korea

**Keywords:** toothbrushing, dental health education, oral hygiene, quantitative light-induced fluorescence

## Abstract

This randomized controlled trial aimed to educate patients and manage oral hygiene remotely via a smart toothbrush (ST) by using mobile applications and to improve oral hygiene and habits by evaluating the plaque state via images from a Q-ray cam, which is a quantitative light-induced fluorescence (QLF) digital imaging device. In total, 68 children (aged 6–12 years) were randomly divided into three groups. Group I was assigned an electro-toothbrush (ET), group II was assigned a smart toothbrush (ST), and group III used a manual toothbrush (MT). Each group used an assigned toothbrush and was checked after 1 and 3 months. Oral hygiene status was evaluated using the patient hygiene performance (PHP) index and fluorescent plaque index (FPI), which are presented in the QLF program. In addition, questionnaires on oral health behaviors and attitudes were also evaluated. There was a 0.24 decrease in the PHP index and a 1.40 decrease in the simple hygiene score three months later compared with the baseline in the ST group, with no significant difference between the three methods (*p* = 0.518, *p* = 0.626). Many group II participants said that ST helped with oral hygiene, and they were willing to use it continuously. In addition, all participants’ brushing times and attitudes toward oral hygiene improved after oral hygiene education using a Q-ray cam. Therefore, the use of STs provided good dental health education and a plaque-decreasing effect to children aged 6–12 years old. Furthermore, the QLF device can be used as a useful tool for maintaining good oral hygiene in children.

## 1. Introduction

Maintaining good oral hygiene is essential for preventing the occurrence and progression of common oral diseases, such as dental caries and periodontal diseases [1]. Among several methods to maintain good oral hygiene, self-performed mechanical plaque control, which involves daily tooth brushing, is of the utmost importance [2]. 

Oral hygiene is important from a very young age to prevent early childhood caries, according to the World Health Organization Implementation manual [3]. Furthermore, according to the American Academy of Pediatric Dentistry guidelines, caries-risk assessment and management includes the evaluation of “visible plaque on teeth” from zero to five years of age and at more than six years of age. Furthermore, caries of the pits and fissures of permanent teeth continue to be a problem in children [4]. Therefore, the importance of adequate oral hygiene increases around six years of age, the age at which the first permanent molar emerges in the oral cavity. In addition, children around this age are instructed to brush themselves under parental supervision to make tooth brushing a daily habit and to learn about the concept of oral hygiene maintenance. Tooth brushing habits formed at this time will persist throughout life, without significant changes [5].

Nevertheless, it is well known that the toothbrushing skills of children under 12 years of age are insufficient [6,7,8]. The reason for this insufficiency might be poor manual dexterity, lack of motivation, or difficulty in understanding precise instructions [9,10]. Moreover, children’s concentration when brushing their teeth is not very high; therefore, it would be beneficial to have new materials to attract them.

In recent years, the age of children using smartphones has continuously lowered [11]. Owing to the development of the IT industry, smartphones are becoming an indispensable factor in quality of life and daily life. In line with this trend, the need for oral health education using smartphones is emerging [12]. Nowadays, the use of mobile applications to raise children’s interest in tooth brushing has been devised. These applications record children’s brushing habits and data, allowing dentists to monitor tooth brushing remotely. Dentists can provide feedback via mobile applications, which opens a new chapter in teledentistry.

What can open the door to teledentistry is a special toothbrush, called a smart toothbrush (ST). Children are instructed to brush with the ST while viewing the instruction (rolling method) video on the smartphone’s display [13]. 

Although many studies have investigated the effectiveness of electro-toothbrush (ET) or manual toothbrush (MT) on children’s dental health, studies on ST education for children are lacking. Therefore, the first aim of this study was to evaluate the plaque reduction efficacy of ST compared with other toothbrushes using the plaque index and quantitative light-induced fluorescence (QLF) technology among 6–12 year-old children. Secondly, to investigate changes in toothbrushing habits through toothbrushing instruction with QLF technology by means of a questionnaire. Lastly, this study aimed to assess the educational effectiveness of ST through a subjective survey.

## 2. Materials and Methods

### 2.1. Study Design and Study Setting

From February 2021 to December 2021, a randomized study was performed at Yonsei University Dental Hospital, Seoul, Republic of Korea. Written informed consent was obtained from the parents or caregivers of all participants before the study. The study was performed according to the protocols and procedures approved by the Institutional Review Board of Yonsei University Dental Hospital (2-2020-0082).

### 2.2. Participants

**Figure 1:**
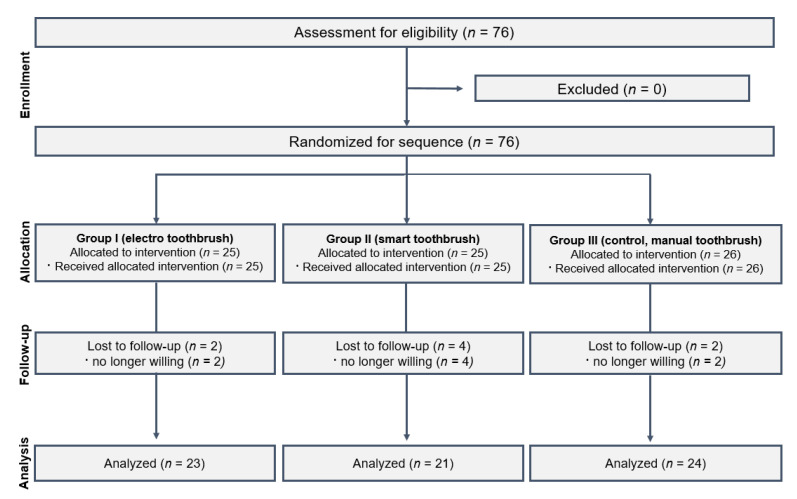
Flowchart of the participants in the randomized clinical trial.

The study’s sample size was calculated using the Gpower program (G*Power Version 3.1.9.4. statistical software). The sample size was estimated to be 16 children in each group, accounting for a total sample size of 48 at a power of 0.80 and an alpha error of 0.05. By taking into consideration a high dropout rate, this study included a sample size of 76 children in total, with 25 or 26 children in each group. In total, 76 children aged 6–12 years old with normal physical and mental development were assessed for eligibility. After excluding participants who withdrew from the study, 68 participants were included in the final analysis and were divided randomly into three groups by lot (Figure 1).

**Figure 2:**
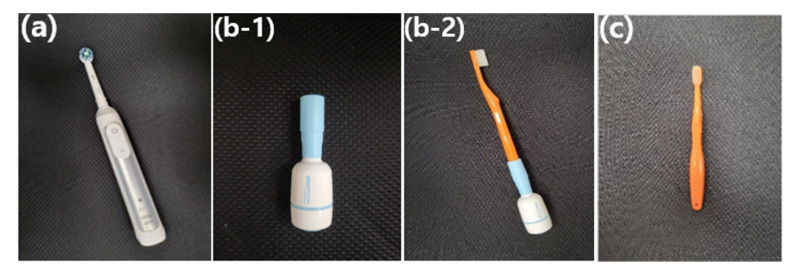
The toothbrushes used in this research. (**a**) electro-toothbrush (Oral-B, Procter and Gamble, Kronberg, Germany); (**b-1**) smart toothbrush machine (XiuSolution, Gyeonggi, Republic of Korea); (**b-2**) machine linked to a manual toothbrush, referred to as a smart toothbrush; and (**c**) manual toothbrush (AIOBIO, Seoul, Republic of Korea).

Group I was assigned to a brush with an ET (Oral-B, Procter and Gamble, Kronberg, Germany), which had a combination of a rotation oscillation movement. Group II brushed with ST (XiuSolution, Gyeonggi, Republic of Korea) that could be attached to the classic toothbrush. Group III used an MT (AIOBIO, Seoul, Republic of Korea) (Figure 2). The total number of subjects were 23, 21, and 24 in groups I, II, and III, respectively. 

**Figure 3:**
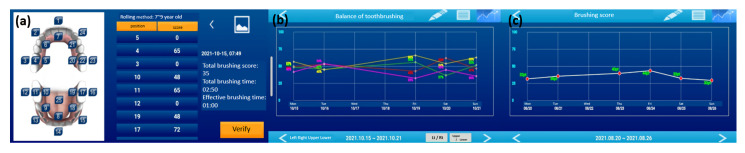
The application of the smart toothbrush. (**a**) The score expresses the brushed area and how well the child brushed. (**b**) The graph shows the upper, lower, left, and right balance of toothbrushing. (**c**) The graph shows the score after brushing teeth every day.

ST has a motion-sensing skill with a 3D accelerometer and magnetic sensor that perceives the location and movement of the toothbrush [14,15,16]. The location and movement data of the ST were converted and analyzed to evaluate the brushing habits and scores via a mobile application (Figure 3).

The inclusion criteria were as follows:

(i) Korean children aged 6–12 years, well-nourished, and in good general health.
(ii) Children with plaque accumulation on the buccal surface on teeth who require tooth brushing instruction (TBI) and good brushing habits.
(iii) Eruption state of permanent first molars and central incisors.
(iv) Children or parents who use android software smartphone.

The exclusion criteria were as follows:

(i) Children with molar–incisor hypoplasia.
(ii) Medically compromised children (neurological disorders and cerebral palsy).
(iii) Children or caregivers who withdrew their consent.

### 2.3. Plaque Assessment

Oral hygiene status was evaluated using the Patient Hygiene Performance (PHP) index, with the scores recorded for six index tooth surfaces: buccal/labial surfaces of both maxillary first molars, the maxillary right and mandibular left central incisors, and lingual surfaces of both mandibular first molars. The PHP index is used to assess microbial biofilms by dividing the tooth surface into the proximal, distal, and middle thirds, and subdividing the middle third horizontally into the incisal, middle, and gingival thirds [17]. Here, the PHP index was evaluated using a quantitative light-induced fluorescence (QLF) device instead of the application of a disclosing agent, because the application of a disclosing agent would have a masking effect when the participant used a Q-ray cam. Each area was scored one point if the colored area persisted, and zero points if it did not. For a single tooth, the score could be zero to five, and the sum of all six teeth was divided by the number of measured teeth to derive the mean. 

**Figure 4:**
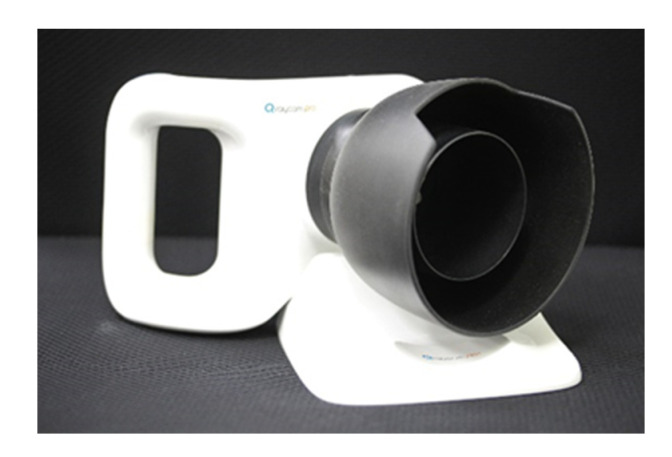
Q-ray cam, quantitative light-induced fluorescence device (AIOBIO, Seoul, Republic of Korea).

Oral hygiene status was also evaluated using a QLF device. Recently, QLF technology has been introduced. By using QLF technology, dental plaques can be visualized as red fluorescence emitted from porphyrins, which are bacterial metabolites inside the biofilm [18,19,20]. By using this technology, a portable camera-type device called a Q-ray cam (AIOBIO, Seoul, Republic of Korea) (Figure 4) was developed so that patients could easily detect dental plaque by themselves. The Q-ray cam can be used in oral health education to provide visual feedback and motivation to children. Several recent studies on oral health education have shown that QLF technology could be useful for improving the oral hygiene status and attitude toward oral hygiene of children and adolescents [21].

**Figure 5:**
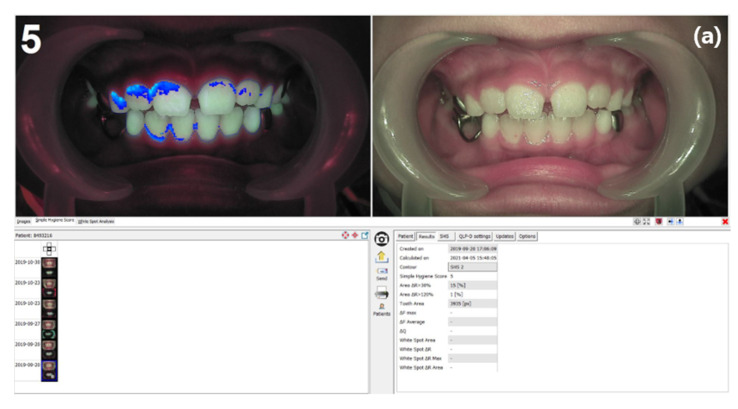

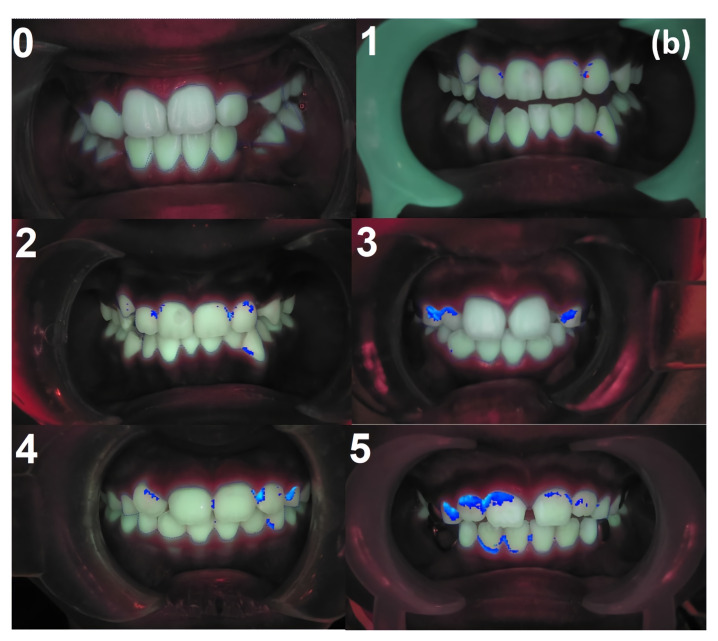
(**a**) Fluorescent plaque index (FPI) analysis using propriety software of the quantitative light-induced fluorescence system. (**b**) Representative fluorescent images with the simple hygiene score (SHS) in the left upper corner. Quantitative and qualitative assessments of dental plaque deposits were performed, and scores ranging from zero to five points were assigned according to the attached area of dental plaque.

QLF images of the frontal, left, and right views were acquired using a Q-ray cam. Fluorescent plaque index (FPI) scoring was automatically performed using the simple hygiene score (SHS) and ΔR30 and ΔR120 values using an analysis program (Q-ray™ v 1.42; Inspektor Research Systems BV, Amsterdam, The Netherlands). The SHS scores the plaque levels as one of six categories (from zero to five) depending on the red fluorescent plaque areas [22]. The color of the sound dentin is green (G) and the plaque biofilm color is red [R], where ΔR is a sub-score of FPI, and it is a measure of the thickness, strength, and maturity of the dental plaque. In this study, ΔR30 and ΔR120 values were measured, and the ratio of the area with increased values was 30.0% and 120.0%, respectively, compared with information in the normal region shown [23]. The average value after adding all of the scores of the frontal, left, and right views was used in the data (Figure 5).

### 2.4. Clinical Procedures 

On the first day of the visit, all participants and parents completed a questionnaire survey on oral health behavior at baseline, which included questions about selecting the number of times teeth were brushed per day, brushing time, and the degree to which oral hygiene is a concern for themselves. The survey questions were modified from the questionnaire developed by Angelopoulou et al. [19].

All participants were also given fluoride-containing toothpaste and randomly assigned to ET, ST, or MT groups during the entire experimental period. A one-to-one TBI lesson was provided to each participant. Afterward, all participants were photographed using a Q-ray cam and brushed their teeth by themselves for three minutes with the assigned brush. All three groups used the rolling method. Thereafter, their teeth were checked once more using the Q-ray cam, and areas with remaining plaque were confirmed by a dentist. For the ST group only, the dentist sent feedback via the application once a week depending on the time, condition, and score of the teeth that could be received via the application.

**Figure 6:**
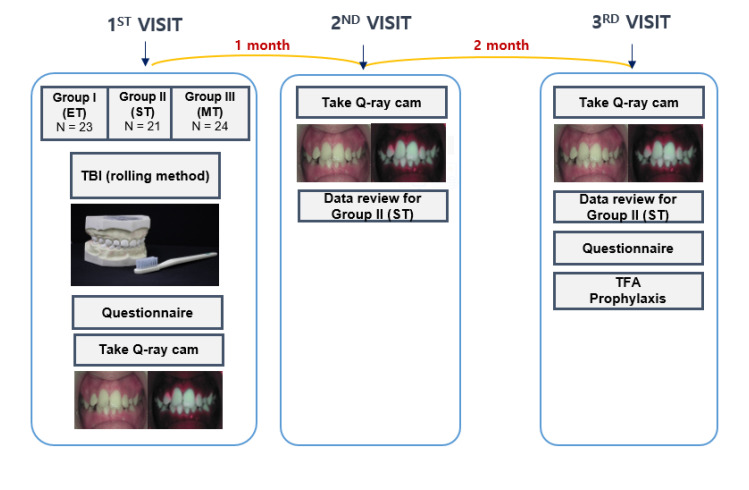
Graphic diagram of this study. ET: electro toothbrush; ST: smart toothbrush; MT: manual toothbrush; TBI: tooth brush instruction; TFA: topical fluoride application.

One month later, participants revisited the hospital for follow-up. Frontal, left, and right views were acquired using the QLF images. In addition, we reviewed the patient’s brushing state. For the ST groups only, we obtained the feedback time by looking at the data so far. Three months later, a final follow-up was performed. The same protocol was performed, and the participants completed a questionnaire about the overall procedures: satisfaction, changes in the number of times teeth are brushed per day, brushing time, and the degree to which oral hygiene is a concern (Figure 6). Eventually, the participants received topical fluoride application (TFA) (varnish type) and prophylaxis with rubber cup polishing.

The procedures were performed by a trained dentist (J.L.) and a dental hygienist (T.L.). The dentist, who was blinded to the brushing method assigned to each child, performed the plaque assessment, and the dental hygienist provided lessons to the participants. To maintain blinding, plaque assessment and tooth brushing were performed in different rooms. 

### 2.5. Statistical Analysis

All of the statistical analyses were performed using the SPSS software version 26 (IBM Corporation, Armonk, NY, USA), with statistical significance set at *p* < 0.05. All variables were confirmed to be normally distributed using the Shapiro–Wilk test. If the normal distribution was not followed, the Wilcoxon signed-rank test was used. Changes between pre- and post-intervention outcome measures were analyzed using a paired sample *t*-test. Differences in oral health-related characteristics were analyzed using the chi-squared test and Fisher’s exact test. The mean PHP, SHS, ΔR30, and ΔR120 were compared among the three groups using repeated-measures analysis of variance (ANOVA).

## 3. Results

Among the 68 children, 39 were males and 29 were females. Groups I, II, and III included 14 males and 9 females, 11 males and 10 females, and 14 males and 10 females, with an average age of 8.65 ± 2.35 years, 9.00 ± 1.51 years, and 8.25 ± 1.59 years, respectively (Table 1).

The PHP score decreased significantly at the three-month check compared with the baseline in both the ET and ST methods (*p* < 0.05). SHS decreased significantly after brushing in all three groups (Table 2).

In the repeated-measure ANOVA, there was a significant difference between time points (*p* < 0.05) in the PHP score and SHS. However, there was no significant difference between the times in ΔR30 and ΔR120.

Furthermore, there was no significant difference between groups and the interaction between group and time in PHP, SHS, ΔR30, and ΔR120 (Table 3).

The responses from the questionnaire survey on the first and last visits are presented in Table 4. There was no significant difference in the number of times teeth were brushed per day, with >75.00% (n = 51) of participants answering two or three times. Among the responses about the brushing time, there was a significant decrease in “less than 2 min” from 14.71% (n = 10) to 4.41% (n = 3) and “2 min” from 42.65% (n = 29) to 23.53% (n = 16); meanwhile, there was a significant increase in “3 min” from 35.29% (n = 24) to 55.88% (n = 38). Among the responses regarding the difficulty of brushing, there was no significant difference between pre- and post-intervention.

**Figure 7:**
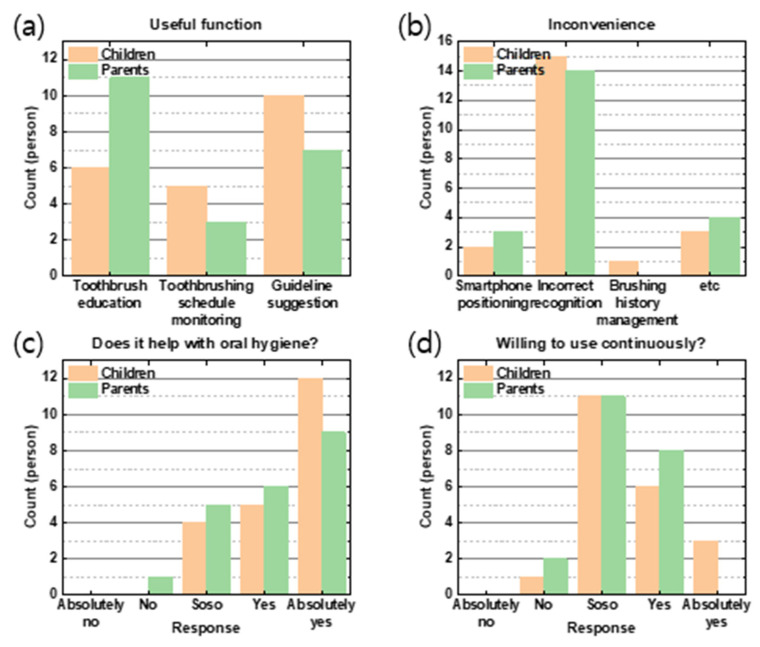
Responses from smart toothbrush users. The orange bar represents children, and the green bar represents parents. The x-axis shows the answers, whereas the y-axis shows the counts of the persons. The following questions were asked: (**a**) “What are the useful functions of ST?”, (**b**) “What are the inconveniences associated with using ST?”, (**c**) “Does ST help with oral hygiene?”, and (**d**) “Are you willing to use ST continuously?”.

Within the ST group (Figure 7), the most preferred function of ST to children was “guideline suggestion”, followed by “toothbrush education.” For parents, “toothbrush education” was the most useful function, followed by a “guideline suggestion”.

The most inconvenient thing about STs was “inaccurate toothbrush recognition” in both groups, followed by scores, hassles, and speed adjustment. The least common answer was “smartphone positioning”. As for whether it helps oral hygiene, “absolutely yes” was the most common response, followed by “yes” and “so so”. The most common intention to use STs was “so so”, followed by “yes” and “absolutely yes”.

## 4. Discussion

With recent developments in children’s smartphone accessibility, the use of STs has become more popular and favorable. A previous study reported that ETs improve motivation by enhancing patients’ interest in oral hygiene, thereby improving their brushing technique [24]. Kim et al. showed that STs have identical effects on plaque control as manual brushes in adults [14]. This study aimed to evaluate the plaque-control ability of STs compared with ETs and MTs in children.

According to the results of this study, group I (ET) and group II (ST) showed significant decreases in PHP scores after three months. Furthermore, SHS measured using the Q-ray program also decreased, suggesting that SHS had a similar tendency as the PHP score. This is consistent with the findings of previous studies that the use of a Q-ray device is effective at detecting the plaque index and improving the oral health status of children in oral health education programs [23,25]. There was a tendency for a decrease in ΔR30 and ΔR120, which are sub-scores of FPI obtained by determining the thickness and maturity of the dental plaque. It is not easier for children to brush their posterior teeth than their anterior teeth; therefore, the values of ΔR30 and ΔR120 were larger for the posterior teeth. In addition, likely, the Q-ray value of the posterior teeth was not accurate because of partial eruption and position, even though both sides of the Q-ray view were observed.

Repeated-measures ANOVA showed that the interaction between time and group was not significant among the three groups. Furthermore, the change in PHP score and FPI of group II (ST) was not significantly different from that of group I (ET) and group III (MT). According to this finding, it is difficult to conclude that using an ST has a better educational effect than the traditional method. Nevertheless, this study is meaningful because children feel that ST helps improve oral hygiene, and the positive response that they would use ST continuously was high. Because oral health habits in children have been shown to establish trajectories that continue into adulthood [26,27], making use of ST as a routine is a good oral health habit for children. In addition, children’s brushing scores and the number of teeth brushed are delivered to dentists through a mobile application; consequently, dentists can regularly obtain information about children’s brushing habits and give feedback through a simple messenger, even if the children do not come to the dentist often. Through this immediate feedback, children’s brushing skills are more likely to grow, and they could maintain clean oral hygiene.

Toothbrushing instructions and feedback conducted at each visit were effective regardless of the type of toothbrush (Table 4). Most participants answered that they brush their teeth twice or thrice daily. This result was similar, regardless of the intervention. Jepsen et al. recommended that the number of times a day to brush their teeth was twice [2], thus indicating that most participants followed the recommendation.

The responses to the brushing times were conspicuous. The answer “three minutes” increased by a large margin following the intervention compared to the baseline. In contrast, the answer “less than two minutes” and “two minutes” decreased. This means that the brushing time of participants increased, and more participants timed the brushing time to 3 min, leading to a reduction in the PHP score. Other studies have shown that plaque scores decreased as tooth brushing time increased [7,8]. A substantial improvement in tooth brushing time in this study could be considered by applying the Q-ray cam to children during the training process to brush by themselves for a three-minute time limit. By informing them that there are still areas where the plaque has not been removed even after three minutes of brushing, participants would have been aware that they should spend enough time brushing their teeth.

However, the number of children who said that it was rather difficult to brush increased. It can be assumed that they felt it was more difficult because they learned how to do it more meticulously. In addition, ST is said to be helpful for oral hygiene; however, there were many cases of “so so” who responded to use continuously; therefore, inconvenience seemed to have influenced it a lot. However, there were many drawbacks, and incorrect recognition was the most resolved. It would be better if technological advances improve inaccurate tooth recognition.

Although there were some weaknesses, there were many advantages, such as no time limit due to the high accessibility of mobile oral health education applications, and continuous, repetitive, and long-term learning is possible [28]. ST is a good alternative to address the limitations of offline oral health education and opens a new chapter in teledentistry. In addition, ST users can obtain feedback from a dentist once weekly; therefore, children can form a good relationship internally, maintain a good relationship with the dentist even during regular checkups, and the dentist can approach them with the concept of a dental home and a personal doctor. ST informs children about the areas they need to focus more on via scores, regardless of whether they brushed, and shows brushing habits that need to be improved via objective figures and graphs. Therefore, ST should play a big role in creating lifelong oral habits in children by utilizing their strengths and improving their weaknesses.

This study had several limitations. First, owing to COVID-19, many Korean kindergartens and elementary schools forbid tooth brushing during lunchtime. As a result, the score may have been affected by the amount of dental plaque accumulated immediately after eating, rather than the usual brushing habits of children who visited the dental clinic immediately after eating or the usual brushing habits of children who visited the dental clinic immediately after school. Second, the Q-ray cam was characterized by a better view of red fluorescence when viewed in a dark environment; therefore, red fluorescence was not easily observed in bright clinics during the daytime. Furthermore, it was not easy to distinguish plaques from the dental cavity because cavities also emit fluorescent substances. Sometimes, the gingiva is mistaken as plaque. Finally, as each child and parent has different interests in oral hygiene and the ability to brush teeth is different, it would have been more meaningful to design a crossover study rather than separate the groups. In addition, the small number of participants was finally included and the difficulty in observing long-term educational effects was due to short observation periods. Therefore, subsequent studies should recruit a wider sample and use a stricter study design to validate the results of this study. There are sex differences in learning ability in dental health education [29,30]; therefore, it will be meaningful to compare the dental plaque indices of male and female child ST users. Furthermore, a follow-up study in which the role of STs can be connected not only to plaque control but also to caries control would be monumental. 

## 5. Conclusions

The use of STs provided good education and a plaque-decreasing effect among children aged 6–12 years. Additionally, the QLF device can be used as an auxiliary tool to motivate children in oral hygiene education. Furthermore, parents encouraged children to brush with an ST, and they were also satisfied with using an ST. In this study, the children’s brushing time and attitudes toward oral hygiene improved after oral hygiene education. Therefore, an ST is an important method for creating lifetime tooth brushing habits. 

## Figures and Tables

**Table 1:** Comparison of the general characteristics of the participants in the three groups.

Gender	Group I	Group II	Group III
Male (number (%))	14 (60.9)	11 (52.4)	14 (58.3)
Female (number (%))	9 (39.1)	10 (47.6)	10 (41.7)

**Table 2:** Variables of FPI in the three methods.

Variables	Group	Baseline	One-Month Check	Three-Month Check	*p*-Value
PHP score	Group I	0.60 ± 0.59	0.58 ± 0.52	0.36 ± 0.37	0.046 *
	Group II	0.67 ± 0.73	0.50 ± 0.59	0.43 ± 0.70	0.046 *
	Group III	0.51 ± 0.77	0.24 ± 0.34	0.39 ± 0.64	0.320
SHS	Group I	2.35 ± 1.95	1.96 ± 1.55	1.42 ± 1.49	0.017 *
	Group II	2.43 ± 1.81	1.46 ± 1.51	1.03 ± 1.09	0.012 *
	Group III	2.29 ± 1.92	1.17 ± 1.44	1.11 ± 1.31	0.001 *
ΔR30 (%)	Group I	11.78 ± 13.70	14.43 ± 15.19	10.39 ± 15.19	0.679
	Group II	11.33 ± 14.04	6.10 ± 8.70	9.43 ± 13.93	0.144
	Group III	14.00 ± 27.26	5.21 ± 7.34	13.38 ± 36.74	0.413
ΔR120 (%)	Group I	3.09 ± 5.29	3.00 ± 5.11	2.30 ± 5.37	0.343
	Group II	2.33 ± 5.13	1.10 ± 2.47	1.71 ± 2.92	0.372
	Group III	4.33 ± 13.07	0.67 ± 1.69	6.21 ± 25.22	0.158

Data are presented as mean ± SD for continuous variables. PHP: Patient Hygiene Performance; SHS: simple hygiene score. * Statistically significant at *p* ≤ 0.05.

**Table 3:** Time effects on variables of oral hygiene status in the three groups.

	Source	*p*-Value
PHP score	Time	0.020 *
	Time × group	0.291
	Group effect	0.518
SHS	Time	<0.001 *
	Time × group	0.339
	Group effect	0.626
ΔR30 (%)	Time	0.238
	Time × group	0.185
	Group effect	0.799
ΔR120 (%)	Time	0.303
	Time × group	0.366
	Group effect	0.716

* Statistically significant at *p* ≤ 0.05. PHP: Patient Hygiene Performance; SHS: simple hygiene score.

**Table 4:** Answers on questionnaire survey at baseline and post-intervention for participants.

Question	Answer	Baseline		Post-Intervention		
		**N**	**%**	**N**	**%**	***p*-Value**
Number of times teeth are brushed per day (n)	1	16	23.53	17	25.00	0.762 ^†^
	2	38	55.88	34	50.00	
	3<=	14	20.59	17	25.00	
Brushing time (min)	Less than 2	10	14.71	3	4.41	0.018 *, ^‡^
	2	29	42.65	16	23.53	
	3	24	35.29	38	55.88	
	4	4	5.88	9	13.24	
	More than 4	1	1.47	2	2.94	
Difficulty of brushing	Very hard	1	1.47	4	5.88	0.366 ^‡^
	Hard	23	33.82	19	27.94	
	Little hard	44	64.71	45	66.18	

* Statistically significant at *p* ≤ 0.05. ^†^: Chi-squared test, ^‡^: Fisher’s exact test.

## Data Availability

Data are available upon request of the corresponding author.
